# Editorial: Machine learning and applied neuroscience, volume II

**DOI:** 10.3389/fnbot.2025.1757770

**Published:** 2026-01-20

**Authors:** Wellington Pinheiro dos Santos, Vincenzo Conti, Orazio Gambino, Ganesh R. Naik

**Affiliations:** 1 Federal University of Pernambuco, Recife, Brazil; 2 University of Enna Kore, Enna, Italy; 3 University of Palermo, Palermo, Italy; 4 Torrens University Australia, Adelaide, SA, Australia

**Keywords:** artificial intelligence, machine learning, neural networks, neuroengineering, neurorobotics, neuroscience

The convergence of machine learning (ML) and applied neuroscience continues to accelerate, driven by the synergistic demands of intelligent systems and deepening insights into the human nervous system. Building upon the success of *Machine learning and applied neuroscience: volume I*, this second volume brings together cutting-edge research that exemplifies how computational intelligence—particularly deep learning, self-supervision, and generative modeling—can address complex challenges in neurorobotics, neurorehabilitation, and behavior-aware intelligent systems.

The four contributions in this Research Topic span a compelling spectrum: from the diagnosis of gait dysfunction in stroke survivors using cost-sensitive classifiers, to the generation of lifelike 3D human motion through generative adversarial networks (GANs), to next-generation sequential recommendation systems that model multi-granularity behavior and feature interactions. Though seemingly diverse, these works share a unifying vision: leveraging advanced ML not only to model neural or behavioral data more accurately, but to extract clinically or functionally meaningful signals that empower real-world applications.

One axis of innovation lies in clinical decision support through interpretable and robust ML. In their study, “*Machine learning-based gait adaptation dysfunction identification using CMill-based gait data*,” Yang et al. tackle gait adaptation dysfunction (GAD)—a pervasive yet under-assessed impairment in post-stroke patients. Using data from an augmented-reality CMill treadmill, they extract kinematic and adaptability features across four ecologically valid tasks (e.g., obstacle avoidance, slalom walking). Among five classifiers evaluated, the AdaCost algorithm—designed to handle class imbalance and misclassification costs—achieved the best sensitivity (80%) and AUC (0.75). Crucially, feature importance analysis revealed that obstacle avoidance success and gait speed were the top predictors, aligning with clinical intuition and offering actionable biomarkers for rehabilitation planning. This work demonstrates how thoughtful integration of domain-aware data collection and cost-sensitive learning can yield deployable diagnostic aids.

Parallel advances emerge in synthetic data generation for human-motion understanding. Wang et al., entitled “*3D human pose data augmentation using Generative Adversarial Networks for robotic-assisted movement quality assessment*” introduce a novel GANs–SVM–DenseNet pipeline to augment 3D human pose datasets—addressing a persistent bottleneck in training data scarcity and limited motion diversity. Their framework uses robotic-assisted capture for high-fidelity grounding, GANs to generate realistic and varied motion sequences, DenseNet for hierarchical feature extraction, and SVM for precise motion-quality classification. Evaluated across four benchmarks (Human3.6M, MPI-INF-3DHP, NTU RGB+D, HumanEva), the model outperforms state-of-the-art methods in both accuracy (>96% on Human3.6M) and efficiency (30% faster inference). By closing the loop between data synthesis, feature learning, and quality assessment, this approach paves the way for scalable, robot-in-the-loop systems in sports science, rehabilitation, and virtual reality.

Complementing these human-centered applications, two articles push the frontiers of sequential modeling in behavior-aware AI, with implications for neuroscience-inspired user modeling. Zhu et al.(a), at “*Multi-granularity contrastive learning model for next POI recommendation*,” propose MGCL (Multi-Granularity Contrastive Learning) for next Point-of-Interest (POI) recommendation. Recognizing that user mobility is expressed not only at the location level but also through regions and categories, MGCL constructs multi-granular sequences and applies contrastive learning to encourage mutual enhancement across granularities. Experiments on three real-world datasets show consistent gains over 11 baselines—validating that modeling collaborative signals across abstraction levels mitigates data sparsity and enriches preference representation. This principle resonates with hierarchical processing in the brain, where sensory inputs are integrated across spatial and categorical scales.

Extending this theme, the same team presents FIDS (Feature Interaction Dual Self-Attention Network) in the article entitled “*Feature Interaction Dual Self-attention network for sequential recommendation*” [Zhu et al.(b)], which challenges the assumption that item features are independent. FIDS first uses intra-item self-attention to model higher-order feature interactions (e.g., between brand, category, and seller in e-commerce), then employs dual self-attention streams to capture sequential dependencies both in item sequences and in the derived integrated-feature sequences. This architecture outperforms strong baselines—including FDSA and SASRec—by up to 9% in HR@5 on the Tmall dataset, proving that explicit modeling of feature interdependence enhances behavioral prediction. Such architectures may inform computational models of cognitive binding, where disparate perceptual attributes are fused into coherent percepts.

Collectively, these works illustrate a maturing field where ML and neuroscience co-evolve: ML architectures grow more neurobiologically plausible (e.g., through hierarchy, attention, and contrastive learning), while neuroscience and clinical applications benefit from increasingly nuanced, robust, and interpretable models.

Looking ahead, key challenges remain—particularly around model explainability, real-time deployment in assistive robotics, cross-population generalizability, and ethical handling of behavioral data. Yet the trajectory is clear: the fusion of machine intelligence and applied neuroscience will continue to yield technologies that are not only more intelligent but also more human-centered.

We thank the authors for their outstanding contributions, the reviewers for their rigorous assessments, and the editorial team at *Frontiers in Neurorobotics* for their support. We hope this volume inspires further interdisciplinary collaboration at the intersection of algorithms, brains, and behavior.

